# Perceived Relationship Benefits of Coresident Caregiving in Parent-Adult Child Dementia Care Dyads

**DOI:** 10.1093/geroni/igaf122.3824

**Published:** 2025-12-31

**Authors:** Courtney Polenick, Kabreah Gunter, Sree Saripalli, Abbie Schoepke, Bhavana Rajesh, Charity Garner

**Affiliations:** University of Michigan, Ann Arbor, Michigan, United States; University of Michigan, Ann Arbor, Michigan, United States; University of Michigan, Ann Arbor, Michigan, United States; University of Michigan, Ann Arbor, Michigan, United States; University of Michigan, Ann Arbor, Michigan, United States; University of Michigan, Ann Arbor, Michigan, United States

## Abstract

The transition to coresident caregiving may be both challenging and rewarding for people living with dementia (PLWD) and the adult child care partners who support them. However, we currently know little about how this transition can impact the parent-adult child relationship from the perspectives of both PLWD and their adult child care partners. In this mixed methods study, we evaluated perceived relationship benefits or improvements following the transition to coresident caregiving among PLWD and their adult child care partners. Participants included 13 people living with dementia (PLWD; M = 84.38 years, SD = 8.30) and their adult child care partners (M = 55.85 years, SD = 5.86) who started coresiding with and supporting the PLWD within the past 5 years. Both care dyad members separately completed recorded phone interviews that included semi-structured questions about how sharing a home and caregiving has benefited or improved the parent-adult child relationship. We conducted qualitative content analysis to determine major themes in the transcribed semi-structured interview data at the dyad level. There were five major themes related to perceived relationship benefits or improvements within care dyads: 1) increased time spent together; 2) mutual exchanges of emotional and practical support; 3) caregiving rewards for both care partners and PLWD; 4) care partner empathy toward PLWD; and 5) resilience in maintaining a close and supportive pre-caregiving relationship. These findings inform targeted interventions to improve well-being among PLWD and their adult child care partners during the critical transition to coresident caregiving.

